# The consequences of effortful emotion regulation when processing distressing material: A comparison of suppression and acceptance

**DOI:** 10.1016/j.brat.2009.05.007

**Published:** 2009-09

**Authors:** Barnaby D. Dunn, Danielle Billotti, Vicky Murphy, Tim Dalgleish

**Affiliations:** a Medical Research Council Cognition and Brain Sciences Unit, 15 Chaucer Road, Cambridge, CB2 7EF, United Kingdom; b Faculty of Education, University of Cambridge, 184 Hills Road, Cambridge, CB2 8PQ, United Kingdom

**Keywords:** Emotion regulation, Suppression, Acceptance, Memory, Emotion, Intrusions

## Abstract

The present study investigated the consequences of different forms of emotion regulation. Eighty nine healthy participants viewed a distressing video of the aftermath of road traffic accidents under either suppression (of both felt and expressed affect), acceptance, or no-regulation control instructions and the immediate and longer-term consequences on emotion, mood, and memory were examined. Suppression (relative to control) led to reduced subjective experience of fear when viewing the video, but did not alter electrodermal (EDA) or heart rate (HR) response. Subsequently, suppression led to a less marked subjective emotional reaction to positive but not negative emotional images, reduced free recall memory of the video, and a greater likelihood of experiencing zero intrusions of the video's content. Acceptance (relative to control) had no impact when viewing the video, was associated with a less marked increase in EDA activity in the 5 min period immediately after viewing the video, a more marked HR deceleration and EDA response to both positive and negative images, and elevated negative affect at one week follow-up. These findings suggest, contrary to the current clinical zeitgeist, that emotion suppression can successfully lead to an ongoing down-regulation of emotion and memory, whereas acceptance may elevate subsequent emotionality.

## Introduction

Central to well-being is the ability to regulate emotions, defined as the automatic and intentional means by which people influence the emotions they have, when they are experienced, and how they are experienced and expressed (Gross, 1989). Effective emotion regulation enables the individual to cope adaptively with a wide range of environmental contingencies but when it goes awry it is increasingly recognised it may have negative emotional and cognitive consequences, potentially becoming a development or maintenance factor in mental or physical ill health (e.g. Berenbaum, Raghavan, Le, Vernon, & Gomez, 2003; Gross & Levenson, 1997; Pennebaker, 1997).

It is therefore important to better understand the positive and negative consequences of different emotion regulation strategies. In this regard, there is ongoing controversy in the field about the consequences of deliberate attempts to suppress emotion, defined here as effortful attempts to down-regulate the internal experience and external expression of unwanted (usually negative) affect. Contrasting predictions emerge from the clinical and normative literature about the consequences of such emotion suppression.

### The ‘maladaptive suppression hypothesis’

On the one hand, the current clinical zeitgeist is to argue that chronic attempts to intentionally reduce emotion via suppression are generally unhelpful, whereas adopting an accepting, non-judgmental stance towards emotions is helpful. So called ‘third-wave’ interventions increasingly advocate the use of mindfulness and/or acceptance techniques (e.g. Acceptance and Commitment Therapy [ACT]; Hayes, Strosahl, & Wilson, 2003; Dialectical Behavior Therapy [DBT]; Linehan, 1993; Mindfulness Based Cognitive Therapy [MBCT]; Segal, Williams, & Teasdale, 2001), which encourage clients to adopt an accepting, observing, non-judgmental relationship to their emotions and other internal phenomena. This importantly differs from standard cognitive therapy approaches in that the focus is no longer on changing the content of thoughts, feelings and beliefs that individuals attach to specific events, but instead changing the *relationship* to this content. Effectively, acceptance involves relinquishing effortful emotion regulation.

This emphasis on acceptance initially emerged from the influential thought suppression literature, which demonstrates the sometimes paradoxical effects of attempts at thought control. It has become almost a clinical truism that intentionally pushing thoughts out of mind results in a paradoxical increase in their frequency either at the time of suppression or subsequently (the ‘rebound effect’)(Wegner, 1994; Wegner, Schneider, Carter, & White, 1987; for reviews see Dalgleish, Hauer, & Kuyken, 2008; Purdon, 1999; Wenzlaff & Wegner, 2000; Rassin, 2005).

The suppression of emotions rather than thoughts presents a slightly different challenge. In the thought domain the goal is to suppress the object (e.g. a thought about a white bear), whereas in the emotion domain the goal is to suppress the emotional reaction to the target (e.g. feelings of sadness) rather than the target itself (e.g. an unhappy memory). The case has been put forward that emotion suppression similarly results in a paradoxical rebound, for example that suppressing the expression of emotion increases physiological responses to emotional material and impairs social functioning and memory (e.g. Gross, 1998b, 2007).

Henceforth the idea that suppressing emotions backfires shall be referred to as the ‘maladaptive suppression hypothesis’. By ‘maladaptive’ we mean that the consequences of suppression jeopardise or impair productive and appropriate functioning for the individual (cf. Cole, Michel, & Teti, 1994).

### The ‘adaptive suppression hypothesis’

On the other hand, a number of theorists increasingly argue that, for healthy individuals at least, material can in fact sometimes be suppressed successfully (Erdelyi, 2006). Indeed, the experimental literature on thought suppression actually presents a more mixed picture than is generally recognised, with thought rebound failing to be found in some scenarios (e.g. Anderson & Green, 2001; McLean & Boomfield, 2007, Rassin, 2005). Further, it has been well demonstrated using directed forgetting, retrieval induced inhibition or think/no-think paradigms that experimental manipulations of suppression in healthy populations reduce subsequent memory of neutral and emotional stimuli (e.g. Anderson & Green, 2001; Anderson et al., 2004; Barnier et al., 2007; Epstein, 1972; for reviews see Anderson, 2007; Depue, Curran, & Banich, 2007; Erdelyi, 2006).

Relevant evidence about the consequences of emotion-, rather than thought-, suppression can be found in the clinical domain. One factor that has been associated with resilience in the face of exposure to extreme adverse events is repressive coping, whereby individuals avoid unpleasant thoughts, emotions and memories (Bonanno, 2004). For example, repressors show relatively low levels of distress and grief for five years after bereavement (Bonanno & Field, 2001; Bonanno, Keltner, Holen, & Horowitz, 1995) and demonstrate better adjustment following childhood sexual abuse, despite being less likely to disclose, relative to non-repressors (Bonanno, Noll, Putnam, O'Neill, & Trickett, 2003). Similarly, the ability to suppress emotion expression has been related to good adjustment following the September 11th terrorist attacks (Bonanno, Papa, Lalande, Westphal, & Coifman, 2004; Seery, Cohen Silver, Holman, Ence, & Chu, 2008). This perspective (henceforth referred to as the ‘adaptive suppression’ hypothesis) fits well with folk theories of emotion regulation in some cultures, for example the British notion of ‘keeping a stiff upper lip’.

Perhaps more controversially a case could also be made that acceptance in some instances might potentially be counter-productive. Relinquishing effortful emotion regulation and instead allowing the emotional response to occur could in some extreme instances expose individuals to overwhelming negative affect that they cannot simply ‘accept’. This might augment rather than diminish concurrent and subsequent emotional and cognitive consequences of the processing of distressing material.

As far as we are aware, unhelpful consequences of the use of acceptance have yet to be empirically demonstrated, however. There is nevertheless some recent evidence suggesting that adopting a self-immersed (first person, field perspective where emotion experience is recounted and held in awareness), as opposed to a self-distanced perspective (third person, observer perspective where emotion experience is reconstrued and reanalysed), leads to impaired emotion regulation (e.g. Ayduk & Kross, 2008; Kross & Ayduk, 2009). Arguably, acceptance can be seen as closer to self-immersion than self-distancing, since it involves a ‘full and open’ embrace and observation rather than a distanced reanalysis of internal phenomena, and might therefore sometimes be counter-productive as an emotion regulation strategy.

### Laboratory investigations of emotion regulation strategies

Given the increasing promotion of acceptance, and rejection of suppression, especially in clinical practice, it is important that experimental evidence is generated in the laboratory to examine these different strategies in both healthy and clinical populations. In particular, it needs to be established whether the use of emotion suppression, relative to acceptance, results in increased or decreased memory for the emotion-eliciting material and a subsequent growth or reduction in emotional responsiveness. As well as being of theoretical interest, such research will provide a better evidence base for advising clinical populations and/or healthy individuals frequently exposed to negative emotional stimuli (e.g. emergency service personnel) about how most effectively to manage negative emotions. To date, while there has been extensive laboratory investigation of the consequences of suppression of cognitive mental material (e.g. thoughts and memories; Anderson et al., 2004; Dalgleish & Yiend, 2006; Wegner et al., 1987), there has been surprisingly little well controlled laboratory work on the consequences of emotion suppression (Campbell-Sills, Barlow, Brown, & Hofmann, 2006).

The dominant approach has been to compare the consequences of following instructions to regulate emotions in a variety of ways when viewing negative emotional stimuli. A series of well controlled studies by Gross and colleagues have contrasted the emotional, physiological, mnemonic and social consequences of reappraisal (construing a potentially emotion-eliciting situation in a way that reduces its emotional impact) versus suppressing emotion *expression*. Partially consistent with the maladaptive suppression hypothesis, expression suppression, while reducing the external expression of emotion, did not reduce negative emotion experience, led to increased physiological responsiveness, and impaired memory for the emotion-eliciting material. In contrast, reappraisal subjectively reduced negative emotion experience (e.g. Gross, 1998a, 1998b; Gross & Levenson, 1993). Similarly, interacting with individuals who are using expression suppression is rated as more stressful than interacting with individuals using reappraisal (Butler et al., 2003).

More complicated to interpret are the mnemonic consequences of expression suppression. Memory is impaired for material where expression suppression has been adopted during encoding, whereas there is no suggestion that reappraisal alters memory (Richards & Gross, 1999, 2000). Arguably, whether this is seen as a good or a bad outcome depends on whether the individual wants to be able to access this material. In some situations (e.g. an emergency service worker who faces a number of stressful situations each day) the case can be made that this memory impairment may actually be advantageous for emotional well-being. This result, however it is evaluated, is also more consistent with the adaptive rather than maladaptive suppression hypothesis, since material is hypo- rather than hyper-accessible following suppression.

### Outstanding questions

These studies have made an important contribution but a number of questions remain unanswered. First, the focus on expression suppression in these experiments may have limited ecological and clinical validity due to its narrow emphasis. For many individuals the goal of suppression is not simply to regulate the external expression of affect but also to exert control over the internal experience of affect. Indeed, it could be argued that expression suppression is not really a form of emotion regulation at all, since it is concerned with displayed rather than experienced effect and studies generally do not show it has any impact on emotion experience (e.g. Gross & Levenson, 1993; Gross, 1998b). In our view, emotion suppression could be more usefully defined in broader terms, focusing on the down-regulation of both the external expression and internal experience of emotion. Importantly, it may be the case that employing this broader type of emotion suppression has stronger, or even quite different, consequences than expression suppression alone.

As far as we are aware no studies of emotion regulation in healthy individuals have yet broadened suppression to include the down-regulation of both externally expressed and internally felt affect and contrasted this to acceptance. While such an approach has been adopted in individuals with mood disorders (Campbell-Sills et al., 2006), this study did not include a no-regulation control condition. This makes it difficult to interpret if the observed negative effects of suppression relative to acceptance were due to suppression being actively unhelpful, acceptance being actively helpful, or some combination of the two. Moreover, very different results may emerge in a healthy as opposed to a clinical population.

Second, the downstream emotional consequences of using suppression and acceptance have not been studied. It remains an open question as to whether individuals will experience elevated or reduced emotion responsiveness in the time period after they have stopped following the emotion regulation instructions. The maladaptive suppression hypothesis would predict that suppression would result in subsequent elevated emotionality (both background mood and response to other emotional stimuli), whereas the adaptive suppression account would argue that suppression might blunt subsequent emotionality. We do not know of any studies that have yet examined the crucial downstream mnemonic and emotional consequences of suppression relative to acceptance.

Third, these studies confined themselves to measuring the explicit memory effects of suppression. A central feature of reactions to negative events is a simultaneous decrease in explicit memory and an increase in intrusive thoughts and images about the events (e.g. Brewin, Dalgleish, & Joseph, 1996; Dalgleish, 2004). It is plausible that emotion regulation strategies may also impact on these intrusive experiences and in ways that differ from their effects on explicit memory.

### Overview of the current study

It therefore remains an open and important question as to whether emotion suppression can be successfully used by healthy individuals to down-regulate experience or whether it results in an unwanted and paradoxical ‘rebound’. To begin to resolve this issue, the aim of the present study was to contrast the consequences of suppression and acceptance, relative to a no-regulation control condition, in healthy individuals. Participants were asked to view a highly distressing video showing the real-life aftermath of road traffic accidents (RTAs)(compiled by Steil, 1996) and now, referred to as the ‘trauma film paradigm’ (Holmes & Bourne, 2008; Holmes, Brewin, & Hennessy, 2004). Suppression instructions asked participants to down-regulate both their external expression and internal experience of emotion using a range of strategies because, as argued previously, we feel that this is the most ecologically valid definition of the construct.

The immediate consequences of the emotion regulation strategies were indexed in terms of changes in self-reported emotion experience and psychophysiological response (heart rate [HR] and electrodermal activity [EDA]). At the end of viewing the film participants were told to stop following the particular emotion regulation instructions. By asking participants to rate their subjective emotion experience 5 min after viewing the video, it was possible to measure immediate mood recovery (cf. Campbell-Sills et al., 2006).

Next, to measure the impact on subsequent emotional responsiveness of the different forms of emotion regulation, participants viewed a series of emotional images and their subjective emotional responses and psychophysiological reactions to those images were measured (cf. Dunn, Dalgleish, Lawrence, Cusack, & Ogilvie, 2004). To examine the cognitive consequences of suppression and acceptance, at one week follow-up memory for the distressing video was assessed via questionnaire. Participants also handed in a diary reporting their experience of intrusions (spontaneously occurring thoughts or images) about the distressing video (cf. Holmes et al., 2004). Finally, participants rated their mood for the week before and the week after viewing the distressing video, to measure any longer-term impact on mood of the emotion regulation conditions.

### Hypotheses

Our global prediction (consistent with the adaptive suppression hypothesis emerging from the normative literature) was that suppression would in fact be a beneficial form of emotion regulation, successfully ‘down regulating’ negative affect. In contrast, we anticipated that acceptance would lead to potentially unhelpful augmentation of negative affect, based on studies showing the negative consequences of emotional immersion (e.g. Kross & Ayduk, 2009). In terms of immediate emotional consequences, we hypothesised that increasing levels of suppression (suppress > control > accept) would prompt a decrease in subjective negative emotion experience while watching the distressing video and a faster recovery of mood in the 5 min after the video finished (Hypothesis 1). Further, increasing levels of suppression (suppress > control > accept) would be associated with a less marked physiological response when viewing the distressing video and a faster return to baseline in the 5 min after viewing (Hypothesis 2). In terms of subsequent emotional consequences, we predicted that increasing levels of suppression would prompt a less marked subsequent increase in emotionality. Therefore, increasing levels of suppression (suppress > control > accept group) should be associated with reduced subjective and physiological emotional response to the affective images (Hypothesis 3) and a reduced (negative) change in mood for the week following the experiment (Hypothesis 4). In terms of subsequent mnemomic consequences, we hypothesised that increasing levels of suppression (suppress > control > accept) would be linked to a reduced frequency and lower distress levels of intrusions about the distressing video content experienced immediately afterwards and in the week following the experiment (Hypothesis 5). Finally, increasing levels of suppression (suppress > control > accept) would lead to *reduced* free recall and recognition memory of the distressing video at one week follow-up (cf. Richards & Gross, 1999; Hypothesis 6).^1^

## Method

### Participants

Eighty nine community volunteers (49 female) were recruited from the Medical Research Council Cognition and Brain Sciences Unit (MRC CBSU) volunteer participant panel. Inclusion criteria included being between 18 and 65 years of age, reporting no clinically significant current mental health history in response to screening questions, having no past history of PTSD (assessed used the Post-traumatic Stress Diagnostic Scale; Foa, Cashman, Jaycox, & Perry, 1997), and falling within the normal intelligence range (assessed using the National Adult Reading Test [NART; Nelson, 1982). Participants were randomly assigned to the emotion suppression (*n* = 29), emotion acceptance (*n* = 30) or no-regulation control conditions (*n* = 30). All participants provided written informed consent prior to taking part in the study. In particular, care was taken to ensure that individuals were fully aware of the potentially distressing nature of the material they would be asked to view. Participants received an honorarium of £5 (approximately U.S. $8 or 6 Euros) per hour for their participation in the project. The study was approved by the local research ethics committee.

### Measures

Participants attended a 2 h appointment where they completed the tasks in the order described below, seated in a comfortable chair in a darkened room.

#### Baseline mood measures and rest task

To provide a baseline estimate of state mood, participants rated how much sadness, happiness, fear, disgust and distress (cf. Holmes et al., 2004) they were experiencing at the current time (rated on a 100 point pen and paper visual analogue scales, ranging from 0, not at all, to 100, extremely) and they completed the Spielberger State Anxiety Scale (STAI state; Spielberger, Gorsuch, Lushene, Vagg, & Jacobs, 1983). To measure baseline longer-term mood, participants repeated the above happiness, sadness, fear, disgust and distress scales and completed a further three mood questionnaires probing how they had felt on average in the week prior to the experiment. The Beck Depression Inventory (BDI; Beck, Ward, Mendelson, & Erbaugh, 1961) was used as a measure of depression symptoms, the Spielberger Trait Anxiety Scale (trait STAI: Spielberger et al., 1983), with instructions modified to reflect experience over the past week rather than in general, was used as a measure of anxiety symptoms, and the Positive And Negative Affect Scale (PANAS; Watson, Clark, & Tellegen, 1988) was used as a measure of positive and negative affect. To provide a baseline estimate of psychophysiological activity, participants were asked to relax for 3 min at the start of the experiment whilst their EDA and HR activity were recorded.

#### Distressing video and emotion regulation instructions

Participants watched a 12.5 min digitised video of real-life footage of the aftermath of five RTAs (Steil, 1996). Each traumatic scene was preceded by a brief commentary that provided context to the accident and described the people involved. Images in each scene included emergency service personnel working to extract trapped victims, injured victims screaming, dead bodies being moved, and body parts among car wreckage, of an emotional intensity similar to that seen in television news broadcasts. Previous studies have used this as an emotion induction and it has been shown to reliably generate high levels of affect but within acceptable ethical boundaries (e.g. Holmes & Bourne, 2008; Holmes et al., 2004; Schartau, Dalgleish, & Dunn, 2009).

At the end of the video, participants rated their subjective emotion experience during viewing (using the same scales as during the rest period, except that in this instance they were computer administered). Psychophysiological responses were indexed in terms of overall mean EDA and HR activity whilst watching the video (see later psychophysiology method for details of recording protocol). All participants read the following instructions:

“*You will now view the test film. This will show the real life footage of the aftermath from five road traffic accidents, which you are likely to find upsetting. It is important for the experiment that you watch the film, but if you become so distressed that you wish to stop the film let the experimenter know by saying ‘stop’ and we will terminate the experiment. Remember to pay attention to the film and do not look away from the screen, as we will ask you questions about it afterwards. After the video we will ask you to rate how you are feeling.*”

In addition, participants in the suppress condition were told to try and suppress their internally felt and externally expressed emotional responses to the film:

“*It is very important for the experiment that when you watch the film you try and suppress any emotional responses to it you are having. What we mean by this is that you should adopt a detached and unemotional attitude as you watch the film. Try to think about what you are seeing objectively in such a way that you don't feel anything at all. Further, if you do have any feelings try not to let these show and keep a 'straight face'. In other words, as you watch the film, try to behave in such a way that a person watching you would not know that you were feeling anything. For example, if the film makes you feel afraid, we would like you to decrease the intensity of fear that you feel and show.*”

These instructions were based on scripts used by Campbell-Sills et al. (2006) that asked participants to suppress both internal experience and external expression, but additionally adding the instruction to detach from their emotional experience.^2^ In contrast, participants in the accept condition were told to immerse themselves in the film and allow themselves to internally experience and externally express any emotions it produced:

“*It is very important for the experiment that when you watch the film you try and accept any emotional responses to it you are having. Immerse yourself in the film, allowing yourself to internally experience and externally express any emotions it produces. Rather than trying to control your reaction imagine your emotion is like a cloud passing in the sky - a natural phenomena that comes and goes regardless of any attempts you make to influence it. Let the feelings wash over you, being aware of how they make you think, feel and react. Just observe all the different aspects of how you are feeling in response to the film, rather than judging whether the emotion is 'good' or 'bad' or 'wanted' or 'unwanted'. For example, if the film makes you feel afraid, allow yourself to openly feel your fear and show your fear in your face and body. If you do find yourself beginning to evaluate or control your emotional reaction that is fine - just notice you have done it and then gently move your attention back to observing and accepting your feelings in response to the film.*”

These instructions asked participants to accept both the internal experience and external (bodily) expression of emotion to ensure they were a mirror image of the suppression instructions and because conceptual definitions of acceptance emphasise openness to thoughts, feelings and bodily sensations.^3^

Participants in the no-regulation control condition were not given any emotion regulation instructions.

Participants practiced following the instructions whilst viewing a short training film (an excerpt from ‘A Tale of Two Cities’; a mildly distressing documentary about the aftermath of the Nagasaki and Hiroshima bombings) and then viewed the test film. When the film was finished, participants were told they could stop following the emotion regulation instructions and then rated their mood. Full instructions and a copy of the narratives accompanying each individual scene are available from the corresponding author. The film footage is not in the public domain, due to copyright and confidentiality issues.

#### Recovery period

Immediately after viewing the film, participants completed a 5 min auditory stream of consciousness filler task (cf. Wegner, Erber, & Zanakos, 1993) and then again rated their mood (using the same pen and paper scales as described for the rest task), intended as a measure of mood recovery (cf. Campbell-Sills et al., 2006). To index intrusion frequency in the recovery period as a function of condition, the number of times participants spontaneously mentioned the content of the distressing video during the SOC task was also computed.

#### Compliance measures

To measure self-reported compliance with the emotion regulation instructions during the distressing video, at the end of the testing session participants were asked to rate the extent to which they attempted to suppress their emotions, using a visual analogue scale ranging from 0 (not at all) to 100 (extremely)(cf. Schartau et al., 2009). We expected the suppress group to report increased suppression efforts and the accept group to report reduced suppression efforts, relative to the control group. In addition, we measured the use of other emotion regulation strategies during the video. Participants rated the extent to which they tried to change the meaning of the material (a measure of reappraisal) and looked away from the screen or deliberately thought of others things (measures of distraction), using visual analogue scales.

It is also possible that habitual tendencies to regulate emotions may override or interact with Condition in the current experiment, particularly in the control group who were given no clear emotion regulation instructions. To examine this possibility participants completed the Emotion Regulation Questionnaire (ERQ; Gross & John, 2003), a measure of the habitual use of expression suppression and reappraisal. The relationships between ERQ factor scores and reported efforts to suppress, look away, deliberately think of other things, or reappraise were then examined in each group separately.

#### Affective picture task

To measure the impact of emotion regulation condition on subsequent processing of emotional material, participants were shown fifty images predominantly selected from the International Affective Picture Set (IAPS; Lang, Greenwald, Bradley, & Hamm, 1993) approximately 5 min after viewing the distressing video (10 positive, 10 neutral, 10 sad, 10 disgusting and 10 neutral). The IAPS is a series of emotional and neutral images that have detailed normative ratings and psychophysiological response data (for a review, see Bradley, 2000). The images are as reported in Dunn et al. (2004) (all drawn from the IAPS), with the addition of ten disgusting images drawn from the IAPS and other sources.

Participants viewed each image for 6 s and rated its valence (on a 9 point sliding visual analogue scale ranging from 1 very unpleasant to 5 neutral to 9 very pleasant). There was then an 8 s inter-trial interval before the next image (based on timings used by Bradley, Codispoti, Cuthbert, & Lang, 2001). Participants were instructed to view the pictures as they naturally would and were not given any particular emotion regulation strategies. They were then told to rate how the image made them feel as honestly as possible. To control for order effects, the images were presented in a pseudo-random sequence. On each block of five trials, one of each image type would be randomly selected, presented, and then excluded from selection on subsequent trials in that block. This ensured that one exemplar of each image type was displayed every five trials. The task took around 30 min to complete on average.

Electrodermal and HR response to each image were also recorded (see psychophysiology methods). HR response to each image was indexed by subtracting mean activity in the 1s period prior to viewing each image from mean activity for the duration of the picture presentation. EDA response to each index was calculated by subtracting mean activity in the 1s period prior to viewing each image from the *maximum* level of activity that occurred during the picture viewing period, effectively a measure of peak amplitude. Median EDA and HR response to each picture type for each participant were then computed. EDA data were natural log transformed to correct for a positive skew in the distribution.

Preliminary analyses revealed no interactions between condition and subjective or physiological responses to the sad, fearful and disgusting image types, so in the analyses presented below they are pooled into a composite negative category. Full instructions and the IAPS number of the images used are available from the corresponding author.

#### Intrusions diary

To assess the impact of the emotion regulation conditions on the experience of intrusions about the video content, participants were asked to complete a daily intrusion diary for one week after viewing the video (cf. Brewin & Saunders, 2001; Holmes et al., 2004; Schartau et al., 2009). Intrusions were defined as spontaneously occurring (not deliberate) memories or images of the film. One diary page was provided for each day of the following week, divided into spaces for morning, afternoon, evening, and night. For each intrusion experienced, participants were asked to record the number of times it occurred; whether it was primarily a thought, image, or both; a brief description of the content; and finally a rating of how much distress it caused them (ranging from 0 not at all distressed to 100 extremely distressed). For all times where participants did not experience any intrusions they were asked to enter a zero, to make it possible to be certain participants had completed the diary. The total number of intrusions was counted from the diary entries by the experimenter. To ensure compliance with the diary, participants were instructed how to use it at the end of the first testing session and they were a given a cover sheet with full instructions and a completed example of the diary for one day. At follow-up, participants were asked how often they had been unable or forgotten to complete the diary on an 11 point scale (0: not at all true; 10: extremely true; cf. Davies & Clark, 1998).

#### One week follow up

In a 30 min follow-up appointment one week after the experiment, participants again completed the weekly mood self-report scales (BDI, PANAS, and modified trait STAI), making it possible to look at weekly mood change as a function of the experimental condition (suppress, accept or no-regulation control). To measure effects on explicit memory of the Distressing video, participants also completed a recognition memory questionnaire and a cued recall explicit memory questionnaire about the film (cf. Holmes et al., 2004). The cued recall questionnaire was made up of 15 questions about specific details in the film (three from each scene) (e.g. “What colour was the car that was on fire in a field, by a tree, at the beginning of the first scene”). The recognition memory questionnaire was made up of 20 descriptions of traumatic scenes and participants had to judge whether that item had or had not occurred in the film. Half of the items were genuine and half did not occur in the video (e.g. “The baby in a blanket is passed to a paramedic and placed in an ambulance”). Participants' intrusion diaries were also reviewed.

#### Psychophysiology recording

While participants viewed the distressing video and the affective images (presented via one computer), their EDA and HR responses were continuously measured via a BIOPAC™ MP100 system connected to a separate computer running AcqKnowledge 3.7.2 software (BIOPAC., 1997). To measure EDA response two grounded Ag–AgCl EDA electrodes (BIOPAC TSD203 transducer) were secured ventrally on the distal index and middle finger of the non-dominant hand. Participants washed their hands with soap and water prior to electrode attachment and isotonic BIOPAC EDA paste (with a recommended NaCl concentration of 0.05M; Grey & Smith, 1984) was used as the electrolyte. The transducer was connected to a BIOPAC GSR100C module, with the gain set to 5V, the low pass filter set to 1.0 Hz, and both high pass filters set to DC. The EDA signal was transformed into micro-Siemens (μS) units before being analysed. To measure HR, two disposable Ag–AgCl ECG electrodes were placed on the dorsal forearms with clip-on shielded leads attached. Prior to attachment, the electrode sites were cleaned with alcohol wipes. The electrodes were connected to a BIOPAC ECG100B module, with the gain set to 5000, the R wave detector switched off, the filter switched on, and the high pass filter switched off. The ECG waveform was used to estimate HR (in beats per minute: BPM) using the AcqKnowledge ‘Find Rate’ function. As the GSR transducer included a ground cable, no additional ground lead was used for ECG. Data were acquired at 200 samples per second. Data points more than the three standard deviations from the mean were excluded as outliers in all cases.

Participants were asked to remove any jewellery or watches and instructed to move as little as possible during each trial to help minimise movement artefact. Choice of electrode attachment site was based on published research guidelines for EDA (Fowles et al., 1981) and HR (Jennings et al., 1981).

## Results

### Group comparability

The groups were comparable for age and estimated IQ according to the National Adult Reading Test (NART; Nelson, 1982), *F*s < 1, and gender ratio, *χ*^2^ (2, 86) = 1.46, *p* = .48 (see top of Table 1). Further, there were no significant group differences prior to the experiment on any of the visual analogue ratings of mood, the mood questionnaires or psychophysiology activity during the rest period, greatest *F* = 1.74, smallest *p* = .18.

### Instruction compliance

As intended, the three groups significantly differed on self-ratings of suppression effort (see bottom of Table 1), *F* (2, 86) = 18.02, *P* < .001 (suppress > control > accept, post hoc LSD *p*s < .01). There was also a group difference in reappraisal effort, *F* (2, 86) = 3.77, *p* = .03. The accept group reported reappraising significantly less than the suppress group (*p* < .01) and tending to reappraise less than the control group (*p* = .08), suggesting that, as intended, the accept group ‘turned off’ the use of reappraisal. There were no significant differences between the suppress and control groups (*p* = .36). Participants overall reported not looking away from the screen or deliberately thinking of other things during the film (mean ratings for each group < 16 on a 100 point scale) and there were no significant group differences on these ratings, *p*s > .05, suggesting that the emotion regulation conditions did not induce significantly different degrees of distraction.

Importantly, trait emotion regulation tendencies (measured using the ERQ) did not appear to substantially influence instruction compliance. The only significant associations, and these were with an uncorrected level of alpha, were for trait reappraisal to be positively correlated with the use of reappraisal during video viewing (*r* = .36, *p* = .05) and for trait expression suppression to be negatively correlated with the use of thinking of other things during video viewing (*r* = −.46, *p* = .01) for individuals in the suppression group. All other results were non-significant (*p*s > .05). Further, there were no significant associations found in the control group, suggesting that there is not a clear tendency for individuals to revert to habitual emotion regulation approaches in the absence of instruction.

### Distressing video

Our *a priori* hypotheses were examined using a series of repeated measures ANOVAs for each emotion rating/psychophysiology response separately, with Time (rest, film, recovery) as the within-subjects factor and Group (control, suppress, accept) as the between-subjects factor.

#### Self-report

Indicating that the film was a successful emotion induction and that mood gradually recovered afterwards, there was a significant main effect of Time for all emotions, smallest *F* = 29.90, *p*s < .001 (see top half of Table 2). All three time periods significantly differed from one another on all ratings (post hoc LSD *p*s < .03). Negative emotions increased from rest to the film and then decreased during recovery. Conversely, happiness ratings decreased from rest to the distressing video and then increased during recovery.

Our first hypothesis was that suppression would lead to a reduced subjective emotional reaction when viewing the video and a faster recovery of mood immediately afterwards, whereas acceptance would lead to an increased subjective emotional reaction during viewing and a slower recovery of mood immediately afterwards, relative to the control group. Analysis found no significant main effects of Group for any emotion rating, greatest *F* = 1.20, smallest *p* = .31. There was a trend for a significant, albeit uncorrected, Time by Group interaction for fear ratings, *F* (4, 172) = 2.18, *p* = .07, but not for any other emotion rating, *F*s < 1.

To resolve the fear interaction trend, the change from rest to the film, from the film to recovery, and from rest to recovery were analysed separately in three further repeated measures ANOVAs (cf. Campbell-Sills et al., 2006). For the rest to film comparison the trend for an interaction remained, *F* (2, 86) = 2.75, *p* = .07, whereas for the film to recovery comparison, *F* (2, 86) = 2.03, *p* = .14, and the rest to recovery comparison, *F* (2, 86) = 1.42, *p* = .25, the interaction was no longer significant. The groups were therefore pairwise compared for the rest to film comparison. For the suppress and control comparison there was a significant interaction, *F* (1, 57) = 5.12, *p* = .03, for the suppress and accept comparison there was a trend for a significant interaction, *F* (1, 57) = 3.33, *p* = .07, and for the accept and control comparison there was no significant effect, *F* < 1. This suggests that the suppress group show a smaller increase in fear when watching the distressing video than the control group and show a trend in the same direction relative to the accept group. While there were no significant main or interaction condition effects for the other emotion ratings, it is nevertheless important to note that numerically the data are in the same direction as the fear ratings. The suppress group reported less marked disgust distress, and sadness responses to the film, relative to the other groups.

In summary, only partially supporting Hypothesis 1, suppression was associated with less marked subjective experience of fear during video viewing. There were similar, albeit non-significant, patterns for sadness, distress and disgust ratings, relative to the control condition. There was however no elevation in subjective emotional experience during viewing in the acceptance group, relative to the control group, and there were no differences between any groups in terms of mood recovery immediately after the video.

#### Psychophysiology

Our second hypothesis was that suppression would lead to a reduced psychophysiological response when viewing the video and a faster return to baseline immediately after viewing, whereas acceptance would be associated with an elevated physiological response during viewing and a slower return to baseline immediately after viewing, relative to the control group. Seven participants' psychophysiology data (four in the control, two in the suppress, and one in the accept group) were lost due to equipment failure.

The bottom half of Table 2 shows mean EDA and HR activity during the rest period, film, and recovery period for participants in the control, suppress, and accept conditions. Three outliers (more than three SDs from the mean) were excluded from the HR analysis (two in the suppress group, one in the accept group). Results revealed a significant main effect of Time, *F* (2, 75) = 22.92, *p* < .001. Post hoc LSD tests showed that there was no difference between rest and film (*p* = .87), that HR increased from film to recovery (*p* < .001), and that HR increased from rest to recovery (*p* < .001). There was no Time by Group interaction and no main effect of Group, *F*s < 1.

One outlier (in the control group) was excluded from the EDA analysis. Analysis revealed a significant effect of Time, *F* (2, 76) = 34.94, *p* < .001, with post hoc LSD tests showing that EDA activity increased from rest to film (*p* < .001) and increased further from film to recovery (*p* < .001), also meaning that recovery was significantly greater than rest (*p* < .001). There was no main effect of Group, *F* < 1, but there was a trend for a Time by Group interaction, *F* (4, 154) = 2.17, *p* = .08. To resolve this trend interaction, the changes from rest to film, film to recovery, and rest to recovery were analysed separately. The interactions for change from rest to film, *F* < 1, or from rest to recovery, *F* (2, 79) = 1.45, *p* = .24, were not significant, but the interaction for change from film to recovery was, *F* (2, 77) = 3.83, *p* = .03. Pairwise comparisons showed that there was no significant difference between the control and suppress groups, *F* < 1, but that there was a significant difference between the control and accept groups, *F* (1, 53) = 4.77, *p* = .03, and suppress and accept groups, *F* (1, 54) = 7.71, *p* < .01. The accept group showed a smaller increase in EDA activity from film to recovery than did the other two groups.

In summary, failing to support Hypothesis 2, there was no difference between the three groups' physiological responses during viewing. Also inconsistent with Hypothesis 2, the accept group relative to the other two groups, showed a *less* (rather than more) marked EDA increase in the recovery period, relative to the other two groups.

### Affective picture task

**Fig. 1:**
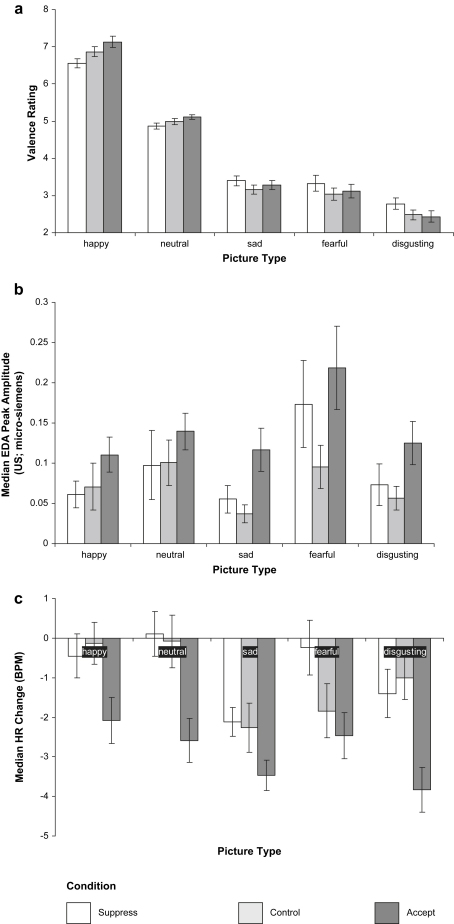
Valence ratings and psychophysiology response to IAPS images. a) Valence Ratings, b) EDA Responses, c) HR Responses. Note – Data are mean (standard error of the mean) values.

Our third hypothesis was that suppression would lead to a less marked subsequent increase, and acceptance a more marked subsequent increase, in subjective and psychophysiological emotional responsiveness, relative to the control group. Fig. 1 shows subjective valence ratings, EDA response, and HR response to each picture type for participants in the three groups. Data were analysed separately for each measure using repeated measures ANOVA, with Picture Type (happiness, sadness, fear, disgust, neutral) as the within-subjects factor and Group (control, suppress, and accept) as the between-subjects factor.

#### Self-report

Valence ratings analysis revealed no significant main effect of Group, *F* < 1, but a significant main effect of Picture Type, *F* (4, 344) = 607.66, *p* < .001. Post hoc LSD tests revealed that positive pictures were rated more positively than all other image types (*p*s < .001), neutral images were rated more positively than all of the negative image types (*p*s < .001), fear and sad images did not significantly differ to one another (*p* = .19), and disgust images were rated more negatively than all other image types (*p*s < .001). There was also a significant Picture Type by Group interaction, *F* (8, 344) = 2.60, *p* = .01.

To resolve this interaction, a series of univariate ANOVAS was run for each image type separately. There was a significant group effect for positive images, *F* (2, 86) = 4.64, *p* = .01, with post hoc LSD tests showing that the suppress group reported lower (less pleasant) ratings than the accept group (*p* < .01), the suppress group tended to report lower ratings than the control group (*p* < .10), and that the control group showed non-significantly lower ratings than the accept group (*p* = .17). There was also a trend for a group effect for neutral images, *F* (2, 86) = 2.58, *p* = .08. Post hoc LSD tests showed that the suppress group reported lower (less pleasant) valence ratings than the accept group (*p* = .03) but that the other groups were not significantly different (smallest *p* = .26). There were no significant group difference for disgust, *F* (2, 86) = 1.78, *p* = .18, sadness or fear, *F*s < 1, images.

#### Psychophysiology

Two participants' psychophysiology data were missing due to equipment failure, leaving 29 participants in each group for the EDA analysis. Analysis found a main effect of Group, *F* (2, 84) = 3.87, *p* = .03, with the accept group showing a greater overall EDA response than the other groups (*p*s < .05), but the suppress and control groups not significantly differing (*p* = .56). There was also a significant effect of Picture Type, *F* (4, 81) = 4.15, *p* < .01, but no Picture Type by Group interaction, *F* < 1. Post hoc tests showed that the fear images showed a greater EDA response than positive, sad and disgusting images (*p*s < .01) and a near-significant greater response than neutral images (*p* = .07). Neutral images showed a greater response than sad images (*p* = .03) and a near-significant greater response than positive images (*p* < .07). No other comparisons were significant (*p*s > .10).

A further five participants were excluded from the HR analysis as outliers (more than three standard deviations from the mean). This left a final sample size of 25 in the control group, 28 in the suppress group, and 28 in the accept group for HR analysis. Overall, participants showed an HR deceleration when viewing the images, consistent with an initial orienting response (Bradley, 2000). Results showed a main effect of Group, *F* (2, 78) = 6.78, *p* = .002. The accept group showed a greater HR deceleration across all image types than the control and suppress groups (post hoc LSD *p*s < .005), whereas the suppress and control groups did not significantly differ (post hoc LSD *p* = .70). There was also a significant effect of Picture Type, *F* (4, 75) = 8.87, *p* < .001. The positive and neutral images produced a less marked HR deceleration than sad and disgusting images (*p*s < .01) and tended to produce a less marked HR deceleration than fear images (*p* < .07). Fear images produced a less marked HR deceleration than sad images (*p* < .01) and tended to produce a less marked HR deceleration than disgusting images (*p* = .06). Sad and disgusting images did not significantly differ (*p* = .11) and neither did neutral and positive images (*p* = .92). There was no significant Picture Type by Group interaction, *F* (8, 152) = 1.90, *p* = .07.

In summary, partially consistent with Hypothesis 3, increasing levels of suppression (suppress > control > accept) led to reduced self-reported emotional response to positive but not negative stimuli. Similarly, acceptance led to elevated EDA and HR reactivity to subsequently presented emotional material. There were however no differences between the suppress and control groups.

### Mood change

Our fourth hypothesis was that suppression would lead to a less marked increase, whereas acceptance would lead to a more marked increase, in negative mood in the week following the experiment, relative to the control group. Scores on the mood questionnaires for the week before were subtracted from scores on the questionnaires for the week after the experiment to generate a change score (see Table 3). Due to a combination of experimenter error (7 participants were not given the mood scales at time one to complete) and four participants not attending the follow-up session, the final sample size consisted of 25 in the control group, 23 in the suppress group and 30 in the accept group.

There were no significant group differences for modified trait anxiety, *F* (2, 75) = 1.64, *p* = .20, modified PANAS positive affect, *F* < 1, or BDI, *F* < 1. There was however a significant group difference on modified PANAS negative affect, *F* (2, 75) = 4.54, *p* = .01. The accept group displayed an increase in negative affect in the week after the experiment and this significantly differed from the decrease shown by the control group (post hoc LSD *p* < .01) and non-significantly differed from the decrease shown by the suppress group (post hoc LSD *p* = .12). There was no significant difference between the control and suppress group (post hoc LSD *p* = .12). Therefore, only partly supporting predictions, acceptance leads to elevated negative affect, but only on some measures, and there were no significant differences between the suppress and control groups.

### Intrusions

Our fifth hypothesis was that suppression would lead to reduced, whereas acceptance would lead to increased, intrusion frequency and distress, relative to the control condition. A number of participants SOC data were lost from the recovery phase due to recording equipment failure, leaving 27 participants in the control group, 24 in the suppress group, and 29 in the accept group. Failing to support predictions, there were no significant group difference in total intrusions during the recovery period, *F* (2, 77) = 1.85, *p* = .17 (see Table 4). Of those participants who completed the diary and attended the follow-up appointment (29 in the control group, 30 in the accept group and 26 in the suppress group), self-reported instruction compliance was generally adequate (mean = 2.24; SD = 1.95; ratings made on an 11 point scale ranging from 0 good compliance to 10 poor compliance) and there was no difference between groups in how well they reported following the instructions, *F* < 1. Again inconsistent with predictions, there were no group differences in diary intrusion frequency, *F*(1, 82) = 2.15, p = .12, or distress, *F* < 1.

Weakly supporting Hypothesis 5 however, a post hoc analysis found a significant difference in the proportion of participants in each group reporting zero intrusions, *χ*^2^ = 13.13, *p* < .01. The suppress group had a significantly greater proportion of participants with zero intrusions than both the control group, *χ*^2^ = 6.58, *p* = .01, and accept group, *χ*^2^ = 9.29, *p* < .01, but there was no difference between the control and accept group, *χ*^2^ < 1.

### Memory tests

**Fig. 2:**
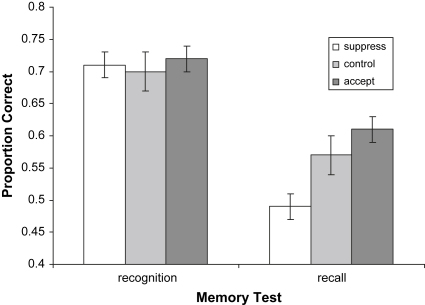
Recognition and recall memory of film content at one week follow-up. Note – Data are mean (standard error of the mean) values.

Our final hypothesis was that suppression would lead to reduced, whereas acceptance would lead to increased, free recall and recognition memory of the distressing video, relative to the control condition. Of the participants who attended the second testing session (28 in the control, 27 in the suppress and 30 in the accept group), there was no significant group difference for recognition memory of the film content, *F* < 1, but there was a significant group effect for free recall of the film content, *F* (2, 82) = 4.89, *p* = .01 (see Fig. 2). Partially consistent with Hypothesis 6, the suppress group had significantly *reduced* free recall memory relative to both the control (post hoc LSD *p* = .04) and accept (post hoc LSD *p* < .01) groups, but there was however no difference between the control and accept groups (post hoc LSD *p* = .33).

### Controlling for trait emotion regulation approaches and the use of reappraisal

Additional analyses were conducted to control for the possibility that trait emotion regulation tendencies could impact on participants' adherence to the experimental instructions and that the conditions were confounded by differential reappraisal effort. All experimental analyses were additionally repeated when entering ERQ reappraisal and expression suppression or reappraisal effort ratings as covariates. In all cases an identical pattern of findings emerged for the key analyses on the main and interaction effects of group in both cases, suggesting that trait emotion regulation tendencies and reappraisal effort are not confounding the current results.

## Discussion

The present study investigated the immediate and longer-term emotional and mnemonic consequences of asking healthy participants to follow suppression versus acceptance emotion regulation instructions while viewing a distressing video, relative to a no instruction control condition. We contrasted predictions emerging from the third wave cognitive therapy literature that emotion suppression might lead to a ‘rebound’ analogous to that seen following thought suppression (‘maladaptive suppression’; e.g. Hayes et al., 2004) with predictions generated from the normative retrieval inhibition and directed forgetting literatures that emotion suppression might sometimes successfully lead to down-regulation of subsequent responses (‘adaptive suppression’; e.g. Anderson, 2007; Dalgleish et al., 2008). We hypothesised that healthy participants would be able to suppress their emotions and that this would be associated with subsequent, potentially helpful, down-regulation. In contrast, we hypothesised that acceptance would be associated with subsequent, potentially unhelpful, augmentation of response in healthy individuals.

### Overview of findings

In terms of immediate emotional consequences, consistent with Hypothesis 1, suppression *reduced* the self-reported subjective experience of fear while viewing the distressing video, relative to the other conditions, although this was a small effect. Sadness, disgust and distress ratings were also non-significantly lower following suppression. There were however no differences between the acceptance and control groups and no group effects in subjective mood recovery in the 5 min period after viewing the video.

Failing to support Hypothesis 2, there were no differences between groups in EDA or HR responses while watching the video. All groups showed a significant increase in EDA from viewing to recovery, but contrary to predictions this increase was *less* marked in the accept group and there were no differences between the suppress and control groups. This suggests that healthy individuals can to some extent successfully suppress their subjective emotional experience, but less so psychophysiological responses. At face value, these psychophysiological findings contrast with previous findings of increased psychophysiological response following expression suppression, relative to reappraisal (Gross, 1998b; Gross et al., 1993). The Gross studies however do not include a neutral control condition and it may be the case that reappraisal lowers psychophysiological response, rather than suppression elevating it. Moreover, there may be different consequences associated with suppression of expression alone versus suppression of both felt and expressed affect.

In terms of subsequent emotional consequences once the emotion regulation instructions were relinquished, as predicted in Hypothesis 3, acceptance was associated with increased EDA and HR reactivity to subsequently presented emotional (both positive and negative) images, relative to both the control and suppress groups. Only partially consistent with predictions, suppression was associated with reduced self-reported valence ratings to positive and neutral, but not negative, images and there were no differences in the psychophysiology responses to the IAPS images between the suppress and control groups. Similarly, partially supporting Hypothesis 4, the accept group reported more negative affect than both the suppress and control groups. However, there were no significant differences between the control and suppress groups and no significant group differences for BDI, PANAS positive affect, or modified trait anxiety. While one might inevitably expect that acceptance should lead to increased emotionality at the time of encoding, that elevations in (predominantly negative) affect persist long after acceptance has been relinquished raise question marks about how adaptive a form of emotion regulation it is.

In terms of subsequent mnemonic consequences, while there were no differences between groups in the mean number of intrusions reported about the film, partially consistent with Hypothesis 5 there were a significantly greater proportion of participants in the suppress group who reported zero intrusions. There were no group differences in the mean distress associated with each intrusion. Partially validating our sixth hypothesis, the suppress group showed reduced free recall (but not recognition) memory of the distressing video at one week follow-up, relative to the other conditions.

This demonstrates that previous findings of reduced memory following suppression in healthy individuals (Richards & Gross, 1999, 2000) can be replicated when adopting a broader definition of suppression that looks at inhibition of both externally expressed and internally experienced affect. We extend these earlier findings by showing that suppression, while not altering overall intrusion frequency, does make it more likely that individuals will experience no intrusions whatsoever. Again, our results are consistent with effective down-regulation following emotion suppression. Whether or not the impaired free recall and reduced experience of intrusions found in the suppression group is seen as a strength or a weakness is a question of perspective. In some instances, for example emergency services workers frequently exposed to traumatic circumstances that they do not wish to overly intrude into their personal lives, reduced recall of the situations (but with no associated elevation of intrusive phenomena) may be beneficial.

Overall, counter to heuristic beliefs promoting acceptance and discouraging emotion suppression in the clinical literature (e.g ACT; Hayes et al., 2004; DBT; Linehan, 1993; MBCT; Segal et al., 2001), these results generally found no clear ‘rebound’ consequences of emotion suppression within a healthy population. In contrast to findings in the thought suppression literature (e.g. Purdon, 1999; Wegner et al., 1987), no increase in negative affect was observed following emotion suppression. On the contrary, where there were significant group differences, these were suggestive of successful suppression and carry over suppression effects, even when participants report relinquishing the strategy, on both mood and memory. Similarly, while other analyses were non-significant, data generally went in the direction of successful suppression. This successful down-regulation of emotion experience does however carry with it potential costs, in that the suppress group reported reduced *positive* emotion experience on the IAPS task. Future research is warranted to examine whether this could relate to the anhedonic symptoms found in depression and related conditions (cf. Kashdan & Steger, 2006).

### Theoretical and clinical implications

These findings have a number of interesting theoretical and clinical implications. Most importantly, they strengthen the claims emerging from the retrieval inhibition and directed forgetting literatures that suppression can be effectively applied in some circumstances (Anderson, 2007), replicating these findings in a more ecologically valid setting.

Clinically, these results suggest that a more nuanced view of the benefits of suppression versus acceptance may need to be adopted in therapy. Rather than globally discouraging the use of suppression, it may be helpful to carry out a cost-benefits analysis with each individual client for each specific situation and set up behavioural experiments (Bennett-Levy et al., 2004) to examine the consequences of emotion suppression. Similarly, the present results suggest that emotion suppression need not necessarily be discouraged in healthy individuals frequently exposed to trauma, such as emergency service workers (see also Mitmansgruber, Beck, & Schüßler, 2008). Building on previous findings questioning the validity of debriefing interventions (e.g. Tuckey, 2007), our results indicate that it may not be necessary to ‘emote’ to adequately process distressing material. Moreover, acceptance under some circumstances may be counter-productive, leading to a subsequent augmentation of negative affect. These data are consistent with repressive coping being associated with resilience following exposure to extreme adverse events (e.g. Bonanno, 2004).

Further, these findings suggest that individuals may choose to adopt particular forms of emotion regulation partly because of the longer-term mood and memory consequences. For example, emotion suppression may be adopted because it reduces subsequent memory of distressing material. We recommend that future emotion regulation studies also include immediate and longer-term emotional and mnemonic measures to make it possible to pick up the subtleties involved with different forms of emotion regulation.

### Future directions

An important next step will be to examine the boundaries of adaptive/effective suppression. It is entirely plausible that emotion suppression, while potentially beneficial in healthy individuals, could become pathological or counter-productive in clinical groups. Indeed, individuals may continue to use emotion suppression even if it maintains and exacerbates their clinical condition because they have experienced it as helpful when well. For example, an interesting future study will be to contrast the consequences of emotion suppression in the same depressed individuals when they are in episode and recovered to test this possibility. Similarly, habitual preferences for particular kinds of emotion regulation may interact with the success of the kind of state manipulations used in the present study. For example, individuals with high versus low levels of experiential avoidance may show different consequences when using suppression (cf. Feldner, Zvolensky, Eifert, & Spira, 2003).

Future work could also systematically explore the behavioural consequences of suppression and acceptance (e.g. willingness or length of time spent engaging with other emotional material), given that a primary goal of acceptance based clinical interventions is to reduce behavioural avoidance. It may also prove fruitful to develop more systematic ways of training participants in emotion regulation strategies (cf. Schartau et al., 2009). In the present study we gave participants the aim of the manipulation (e.g. to reduce the emotions you feel and show), without giving specific directions about how to achieve this. It is possible that even stronger consequences of acceptance versus suppression may emerge following training.

### Limitations

There are a number of potential limitations with the present study. First, we adopted a broad definition of emotion suppression, asking individuals to down-regulate their emotion experience and expression by whatever means, potentially including detachment, expression suppression and other strategies. Similarly, the global definition of acceptance that we adopted included elements of immersion, openness to experience, and non-judgmental observation. While we feel that these broader conceptualisations of emotion suppression and acceptance have greater ecological validity and link well with clinical approaches to emotion regulation, we acknowledge that these definitions depart from aspects of the existing experimental literature in some ways. For example, the process model of emotion regulation (e.g. Gross, 2007) might view the detachment component of our suppression instructions as a form of reappraisal, and as quite distinct from emotion suppression. Similarly, the acceptance clinical literature is not fully explicit about the extent to which acceptance involves taking a self-detached versus self-immersed relationship to internal phenomena (cf. Kross & Ayduk, 2009). It remains possible that different aspects of emotion suppression and acceptance as globally defined here, for example internal versus expression suppression or self-immersion versus self-detachment, have quite different consequences that we are unable to tease apart in the present study.

Second, asking participants to view a film involving real-life footage of the aftermath of road traffic accidents, while having greater ecological validity than some previously used methodologies, is still different from studying naturalistic, personally experienced negative events. There are however ethical issues about exposing participants to more naturalistic stressors and it is difficult to exert tight experimental control in these scenarios.

Third, aspects of the sample recruited may limit generalisability of these findings. Participants were predominantly white middle class and there were more women and men. There is good preliminary evidence that there are cultural (e.g. Butler, Lee, & Gross, 2007) and gender (Garnefski, Teerds, Kraaij, Legerstee, & vandenKommer, 2004) differences in the acceptability and efficacy of different emotion regulation strategies.

Fourth, it is possible that the results observed in the present study are demand effects, whereby participants rated their mood and memory changed because this is how they felt they were supposed to respond. We feel this is unlikely, however given that care was taken not to suggest to participants that acceptance or suppression would be particularly helpful or unhelpful. Finally, the different experimental instructions were not matched for word length (acceptance > suppression > control), although we feel it is unlikely that this has influenced results.

### Concluding remarks

In summary, the present study adds to the existing normative literature on emotion regulation by showing that the longer-term consequences of emotion suppression are an ongoing dampening of reactivity (reduced emotional response to positive material, reduced memory of the material encoded, and greater likelihood of experiencing no intrusions about the memory encoded). This goes against the predictions of the thought suppression literature, which might suggest there would be a ‘rebound’ following emotion suppression analogous to that often seen following the suppression of thoughts (e.g. Wegner, 1994). In contrast, the longer-term effects of acceptance are better characterised by an elevation in reactivity (more negative affect at one week follow-up, greater physiological response to emotional images). Overall, these results are somewhat contrary to the current clinical zeitgeist (e.g. Hayes et al., 2003) and suggest that it is helpful to take a more nuanced view of the costs and benefits of different forms of emotion regulation. Depending on the goals of the individual, it may be that they prefer to use suppression or acceptance in a particular scenario. In lay terms, a small dose of ‘stiff upper lip’ may at times help individuals adaptively cope with aversive situations.

## Figures and Tables

**Table 1:** Demographic characteristics and compliance with emotion regulation instructions.

	Control (*n* = 30)	Suppress (*n* = 29)	Accept (*n* = 30)
Age	32.80 (16.06)	35.66 (14.98)	37.63 (14.83)
Gender	14 F/16 M	18 F/11 M	17 F/13 M
NART estimated full scale IQ^a^	117.25 (6.12)	117.58 (7.76)	117.21 (9.25)
PANAS – positive	34.17 (7.35)	33.90 (4.27)	34.17 (6.17)
PANAS – negative	15.60 (2.94)	14.00 (2.80)	15.63 (5.69)
STAI – trait	36.27 (7.15)	35.03 (7.74)	37.20 (11.90)
STAI – state	32.10 (6.43)	31.21 (5.33)	30.04 (8.20)
BDI	3.97 (2.98)	3.21 (2.78)	4.87 (5.10)
Suppressed emotions	36.58 (28.70)	58.04 (31.73)	15.50 (20.00)
Changed meaning	25.65 (24.57)	31.80 (31.80)	13.90 (18.60)
Looked away	4.03 (4.23)	4.99 (7.04)	5.33 (10.25)
Thought of other things	8.25 (8.90)	15.42 (27.61)	4.67 (8.47)

Note – Data are mean (standard deviation) values except where otherwise noted. NART = National Adult Reading Test; PANAS = Positive Affect Negative Affect Scale; STAI = Spielberger State Trait Anxiety Inventory; BDI = Beck Depression Inventory. All compliance ratings on a 100 point scale, ranging from 0 not at all to 100 extremely.

a 1 participants NART data in the suppression group was not available.

**Table 2:** Subjective emotional ratings and psychophysiological responses during the rest, film, and recovery periods.

		control	suppress	accept
		(*n* = 30)	(*n* = 29)	(*n* = 30)
Happiness	Rest	66.33 (18.10)	66.76 (18.39)	67.84 (23.49)
	Film	3.84 (9.83)	2.56 (3.18)	2.00 (1.84)
	Recovery	23.47 (22.54)	31.34 (25.52)	27.16 (28.31)

Sadness	Rest	9.61 (12.75)	8.60 (15.83)	6.60 (10.88)
	Film	58.00 (28.19)	50.29 (36.79)	61.77 (29.98)
	SOC	39.79 (26.13)	33.85 (31.41)	39.32 (28.84)

Fear	Rest	6.25 (7.55)	7.81 (14.67)	5.06 (11.84)
	Film	33.35 (25.80)	18.25 (23.93)	30.37 (33.06)
	Recovery	18.53 (19.59)	14.01 (16.93)	21.75 (31.36)

Disgust	Rest	3.31 (4.48)	1.82 (3.55)	1.81 (2.37)
	Film	30.36 (28.66)	23.69 (26.95)	26.47 (33.92)
	Recovery	20.39 (20.07)	18.31 (25.63)	18.91 (22.19)

Distress	Rest	4.79 (6.82)	2.51 (2.81)	2.96 (5.23)
	Film	48.65 (33.99)	41.41 (33.23)	55.07 (35.27)
	Recovery	31.51 (27.74)	30.69 (30.96)	35.33 (33.33)

EDA	Rest	6.93 (2.70)	6.98 (3.35)	6.57 (1.96)
	Film	7.39 (2.97)	7.82 (3.68)	7.41 (2.26)
	Recovery	9.09 (3.53)	9.37 (3.77)	8.01 (2.24)

HR	Rest	71.42 (9.83)	71.54 (9.85)	71.77 (9.35)
	Film	72.04 (9.46)	71.75 (9.24)	71.54 (9.02)
	Recovery	80.85 (10.56)	79.27 (16.04)	78.39 (12.91)

Note – Emotions rated on 100 point visual analogue scales, ranging from 0 not at all to 100 extremely; Electrodermal (EDA) activity recorded in μS (micro-siemens); heart rate (HR) recorded in beats per minute (BPM).

**Table 3:** Change in mood in week following experiment.

	Control (*n* = 25)	Suppress (*n* = 23)	Accept (*n* = 30)
BDI	−.52 (2.72)	−.13 (2.20)	−.33 (3.89)
STAI – trait (modified)	−1.96 (4.89)	−1.27 (3.75)	.39 (5.78)
PANAS – positive (modified)	−.96 (8.45)	−1.30 (6.01)	.53 (7.04)
PANAS – negative (modified)	−3.08 (5.61)	−1.21 (3.19)	.30 (3.26)

Note – Data are mean (standard deviation) values.

**Table 4:** Intrusions data during recovery period and for week following experiment.

	Control (*n* = 29)	Suppress (*n* = 26)	Accept (*n* = 30)
Recovery intrusion number	2.22 (.39)	1.58 (1.74)	1.38 (1.29)
Diary compliance	2.36 (1.42)	2.85 (2.84)	2.20 (1.38)
Diary intrusion number	8.17 (10.90)	4.50 (4.81)	5.03 (3.44)
Diary proportion reporting zero intrusions	.07	.35	.03
Diary intrusion distress^a^	24.33 (20.37)	21.24 (19.04)	23.74 (20.61)

Note – Data are mean (standard deviation) values, except where otherwise stated.

a Calculated only for those participants who reported intrusions (*n* = 27 in control group; *n* = 17 in suppress group; *n* = 28 in accept group).

## Footnotes

1 It is important to acknowledge that these predictions could equally well have been framed in the reverse direction, based on the maladaptive suppression hypothesis and beliefs about beneficial effects of acceptance held in the clinical literature.

2 The influential process model of emotion regulation might see these aspects of our instructions as reflecting ‘reappraisal’, and argue that this is quite distinct from emotion suppression (e.g. Gross, 1998a). However, in our view the experimental definition of ‘reappraisal’ used in the Gross studies (“adopt a detached and unemotional stance”; e.g. Gross & Levenson, 1997) is closer to detachment rather how the concept of reappraisal is utilised in the clinical domain. The essential feature of reappraisal in therapy is not detaching from but changing the meaning of a stimulus, for example learning that a panic attack is a sign of anxiety rather than that a heart attack is imminent. Consistent with this analysis, in a recent reappraisal training study (Study 3; Schartau et al., 2009) we have shown that using global reappraisal themes to change the meaning associated with stimuli has different emotion regulation consequences than simply detaching from the stimuli (based on the Gross definition of reappraisal). In the present study we therefore view ‘reappraisal’ as defined by the process model as a subtype of emotion suppression, and distinguish it from clinical reappraisal involving meaning change. To control for the latter form of reappraisal, which we agree is a distinct form of emotion regulation, in our manipulation check we asked all volunteers to rate how much effort they made to change the meanings associated with the video and repeated all analyses when covarying this out.

3 It is debatable to what extent the instruction to immerse oneself in emotion is an essential component of acceptance. Conceptual definitions of acceptance emphasise the ‘active and aware embrace’ of internal phenomena, which can be seen as a form of immersion. The degree to which acceptance involves ‘immersing’ (adopting a field perspective) versus ‘distancing’ (adopting an observer perspective) from internal phenomena is in our opinion not currently clear however in the clinical literature.
